# EHMTI-0012. Comorbid disorders and the quality of life in children and adolescents with primary headache

**DOI:** 10.1186/1129-2377-15-S1-B15

**Published:** 2014-09-18

**Authors:** I Izmailova

**Affiliations:** 1 Neurology Chair, Astrakhan Medicin Academy, Astrakhan, Russia

## Aims and methods

In order to assess comorbid disorders, their impact on the course of headaches, and quality of life (QL) were examined using questionnaires and neuropsychological methods 279 children and adolescents with tension-type headache, 164 with migraine and 60 healthy controls. Parents signed informed consent for their children's survey.

## Results

The spectrum and severity of comorbid psychosomatic disorders (anxiety, depression, autonomic dysfunction, fatigue, insomnia, cognitive dysfunction) depend on the frequency of headache. In patients with chronic headache, especially teenagers, have expressed anxiety, autonomic disorders, mild depression, insomnia, asthenia, and cognitive disorders, often associated with each other. Dissatisfaction with current life situation noted 95,5% of the patients with chronic headache, increased anxiety and depression - 90,9%, reduction in daily activity - 63,3%, reduction of self-control - 54,5%, a reduction of social contacts – 36,4%, worsening of the relation with parents - 31,8%. Quality of life due to the mutual influence of many medical and social factors: the frequency and intensity of headache, severity of emotional disturbances, personality characteristics, family microclimate, level of social adaptation.

## Conclusions

Established reliable relationship of comorbid disorders among themselves and with quantitative indicators of pain, a significant impact on QL of children and adolescents: in the same form of headache the patients with comorbidity had worse indicators of QL, especially physical activity, emotional well-being, social functioning. With the development of integrated rehabilitation programs for children and adolescents with primary headache the need to pay special attention to the identification and correction of comorbid psychosomatic disorders.

No conflict of interest.

